# One-Year Postfracture Mortality Rate in Older Adults With Hip Fractures Relative to Other Lower Extremity Fractures: Retrospective Cohort Study

**DOI:** 10.2196/32683

**Published:** 2022-03-16

**Authors:** Andrea Dimet-Wiley, George Golovko, Stanley J Watowich

**Affiliations:** 1 Department of Biochemistry and Molecular Biology University of Texas Medical Branch Galveston, TX United States; 2 Department of Pharmacology and Toxicology University of Texas Medical Branch Galveston, TX United States

**Keywords:** hip, fracture, mortality, aging, older adults, elderly, mortality risk, electronic health record, EHR, survival probability, postfracture mortality rate, fall, bone, injury, dementia, diabetes, type 2 diabetes, trauma, treatment, comorbidity, mobility

## Abstract

**Background:**

Hip fracture in older adults is tied to increased mortality risk. Deconvolution of the mortality risk specific to hip fracture from that of various other fracture types has not been performed in recent hip fracture studies but is critical to determining current unmet needs for therapeutic intervention.

**Objective:**

This study examined whether hip fracture increases the 1-year postfracture mortality rate relative to several other fracture types and determined whether dementia or type 2 diabetes (T2D) exacerbates postfracture mortality risk.

**Methods:**

TriNetX Diamond Network data were used to identify patients with a single event of fracture of the hip, the upper humerus, or several regions near and distal to the hip occurring from 60 to 89 years of age from 2010 to 2019. Propensity score matching, Kaplan-Meier, and hazard ratio analyses were performed for all fracture groupings relative to hip fracture. One-year postfracture mortality rates in elderly populations with dementia or T2D were established.

**Results:**

One-year mortality rates following hip fracture consistently exceeded all other lower extremity fracture groupings as well as the upper humerus. Survival probabilities were significantly lower in the hip fracture groups, even after propensity score matching was performed on cohorts for a variety of broad categories of characteristics. Dementia in younger elderly cohorts acted synergistically with hip fracture to exacerbate the 1-year mortality risk. T2D did not exacerbate the 1-year mortality risk beyond mere additive effects.

**Conclusions:**

Elderly patients with hip fracture have a significantly decreased survival probability. Greatly increased 1-year mortality rates following hip fracture may arise from differences in bone quality, bone density, trauma, concomitant fractures, postfracture treatments or diagnoses, restoration of prefracture mobility, or a combination thereof. The synergistic effect of dementia may suggest detrimental mechanistic or behavioral combinations for these 2 comorbidities. Renewed efforts should focus on modulating the mechanisms behind this heightened mortality risk, with particular attention to mobility and comorbid dementia.

## Introduction

Recent studies suggest a 17%-25% 1-year mortality rate following hip fracture or its surgical repair in older adults [1-4]. However, hip fracture has rarely been compared against other fracture types in elderly cohorts to understand its specific impact on mortality risk and the unique vulnerabilities associated with it. Since previous studies have suggested that, under specific conditions, fracture location can impact mortality risk [5,6], a comparison of hip fractures against other fracture types may help inform postfracture medical care to target high-risk patient populations more specifically.

This study comprehensively compared 1-year mortality rates following fractures of the hip, upper humerus, and lower extremities to determine whether mortality risks differed by fracture type or fracture region in the elderly population as a whole. In addition to simple comparisons of 1-year mortality rates associated with different fracture types and combinations across elderly cohorts, we also used propensity score matching across cohorts to reduce the impact of confounding factors. We hypothesized that hip fracture would result in greater 1-year mortality rates compared to fractures that did not similarly impact mobility.

This study also examined the extent to which the age-associated comorbidities of dementia and type 2 diabetes (T2D) increase 1-year postfracture mortality rates. Dementia has been identified as a major risk factor for hip fracture [7], is associated with increased mortality risk after hip fracture [8], and is linked to increased postoperative complications following hip fracture repair [9]. Similarly, T2D has been identified as a fracture risk factor [10], with insulin treatment status additionally modifying the region-specific fracture [11,12]. Importantly, both dementia and T2D are associated with increased mortality risk irrespective of fracture [13-15] and, by comparing different fracture types/combinations in individuals with the same comorbidity, we were able to deconvolute each specific fracture’s mortality rate to determine whether dementia or T2D act synergistically with the fracture to exacerbate mortality outcomes.

## Methods

### TriNetX Queries

Data were acquired through queries performed in the TriNetX database (TriNetX, Cambridge, MA, USA) using the Diamond Network on fractures that occurred from January 1, 2010, to December 31, 2019 (queries were last updated the week of March 28, 2021; this update was to capture patients with fractures within these time frames whose records were added later). Follow-up of patients included 2020 data, despite changes to the age-adjusted mortality rate that year [16], to keep the results as complete and updated as possible. The Diamond Network contains electronic medical record (EMR) and medical and pharmacy claims data [17] from over 200 million de-identified patients across the United States and its territories. Patients without an assigned sex were ignored, as those missing this information likely had an incomplete medical record; TriNetX explicitly defines the sex of patients but does not include gender data. Males and females were divided into age cohorts spanning 10-year intervals (60-69, 70-79, and 80-89 years). To minimize the risk of patient identification, the TriNetX database does not report patients’ data once they exceed 90 years of age. Since we were interested in mortality rates within the first year following hip fracture repair, cohorts ended at 89 years of age.

Codes from the *International Classification of Diseases, Tenth Revision* (ICD-10) were used for fracture diagnoses, and patients were identified as having a hip fracture if a code of S72.0, S72.1, or S72.2 was present in their record. Hip fractures were compared to fractures of the upper humerus (S42.2); regions near the hip (lumbar vertebra, S32.0; sacrum, S32.1; coccyx, S32.2; ilium, S32.3; acetabulum, S32.4; pubis, S32.5; ischium, S32.6); nonhip regions of the femur (femur shaft, S72.3; lower femur, S72.4); knee and lower leg (patella, S82.0; upper end of tibia, S82.1; tibia shaft, S82.2; lower end of tibia, S82.3; fibula shaft, S82.4); and talus, malleoli, and foot (medial malleolus, S82.5; lateral malleolus, S82.6; talus, S92.1; metatarsal, S92.3; great toe, S92.4; lesser toe, S92.5). Fracture codes below the hip that were broadly categorized as “other” or “unspecified” were not included. The upper humerus was chosen as a control for hip fracture since both fractures occur at anatomically similar torso-appendage junctures. The other fracture sites impact mobility to varying degrees and were chosen based on reports that mobility limitations play a critical role in the mortality risk of older adults [18,19]. Comparisons were also performed between the hip fracture grouping and each of the other listed individual fracture codes. In a respective query, only 1 fracture event among the pooled fracture types or for the individual fracture type was allowed to occur from 60 to 89 years of age, but we did not actively exclude individuals who had simultaneous, or even subsequent, fractures across, or outside of, our groupings, even though certain combinations are predicted to worsen outcomes [20,21]. In addition, although we removed individuals who experienced multiple fracture events of the same kind or grouping from 60 to 89 years of age to reduce confounding variables, some of these removed individuals may have fractured a different bone under the same code or pool of codes or the same bone contralaterally, and it may not be a repeated fracture of the same type. The analysis did allow for the same fracture to have occurred prior to 60 years of age. The incidence of each fracture type or grouping within the specified decade was initially established across sex and age, and the percentage of individuals in each sex and age cohort deceased from 1 day to 1 year postfracture was determined by dividing the number of individuals deceased by the total population with same-day deaths subtracted. Since the TriNetX database cannot report information for cohorts with 10 or fewer individuals, the number of same-day deaths had to be determined by subtracting the deaths that occurred from 1 day to 1 year from the deaths that occurred within 1 year for the same respective cohort. Same-day deaths were excluded from analyses since these individuals may not represent a population that can be treated postoperatively following a fracture event. To improve readability when reporting data, the term “1-year postfracture mortality” has been used herein to describe those deaths that occurred from 1 day to 1 year postfracture.

The relative frequencies of each fracture were calculated by dividing the total number of incidences of the fracture or fracture grouping by the sum of the incidence for all fractures or fracture groupings and then converting it to a percentage for each respective sex and age cohort. There was a small possibility of counting a patient multiple times if they experienced 1 or more events that fell within multiple fracture groupings.

Since few individuals had unique simultaneous hip fracture combinations in a single event (specifically, combinatorial fractures of S72.0xS72.1, S72.0xS72.2, S72.1xS72.2, and S72.0xS72.1xS72.2), we focused our efforts on incidences and mortality rates for each separate fracture code, excluding the other hip fracture codes for each of these individual analyses. We also included a metric composed of all fracture codes/combinations in the hip. The percentage change in survival probability was determined by subtracting the hip fracture mortality rate from the mortality rate of the other fracture/fracture grouping of interest.

To examine the impact of dementia on fracture outcomes, we explored patients with a diagnosis of vascular dementia (F01), dementia due to Alzheimer disease (G30), dementia with Lewy bodies (G31.83), or any combination thereof; these diagnoses were chosen in order to better unravel potential mechanisms of action since they encompass a large proportion of individuals with at least 1 specified form of dementia while removing individuals with unspecified dementia and relatively rare forms of dementia [22]. Dementia diagnoses were allowed from any time before the fracture to 1 year postfracture (including exactly 1 year postfracture). For T2D diagnosis, the code E11 was used and was required to be present in a patient’s record from 6 months prior to the fracture to 1 year postfracture in order to ensure that patients with reversal or remission of T2D were excluded [23].

General queries determined the sizes of the male and female populations with a dementia or T2D diagnosis from 60 to 90 years of age from January 1, 2010, to December 31, 2019. Unlike the fractures, where we only allowed ICD-10 codes through 89 years of age, these diagnoses were allowed through 90 years of age since we were focused on mortality outcomes and this made them comparable to our mortality measures in our fracture cohorts that had allowed diagnoses of dementia and T2D through the 1-year follow-up. These were plotted using BioVenn [24]. Analyses were run on these subpopulations identically to the overall fractured populations, with the exception that cohorts were not propensity-score-matched because the cohort size was limited by requiring a fracture event in combination with a dementia or a T2D diagnosis.

### Statistics

Propensity score matching was performed across different fracture groupings within the TriNetX system for the root category of each demographic, diagnosis, medication, procedure, and common lab variable (a maximum of 191 broad categories of characteristics outlined in Table S1 of Multimedia Appendix 1) recorded in both cohorts through 1 day before the fracture event. The day of the fracture was not included, since some patients may have received more immediate, or different, care than others. Not all cohort combinations had data for every characteristic. Propensity-score-matching methods can be found in Multimedia Appendix 2. Kaplan-Meier (KM) and hazard ratio (HR) analyses were performed using the matched cohorts of patients, excluding those that died the same day as the fracture. Log-rank tests established the statistical significance of the KM survival curves 1 day to 1 year postfracture. HRs, the CIs for the HRs, and tests for proportionality were calculated within the TriNetX system, which uses the R Survival package (v3.2-13) [25,26] and validates these results by comparing to them to those of SAS (v9.4; SAS Institute). The proportionality test was based on the scaled Schoenfeld residual [27].

## Results

### Fracture Incidences, Relative Frequencies, and 1-Year Mortality Rates

**Figure 1:**
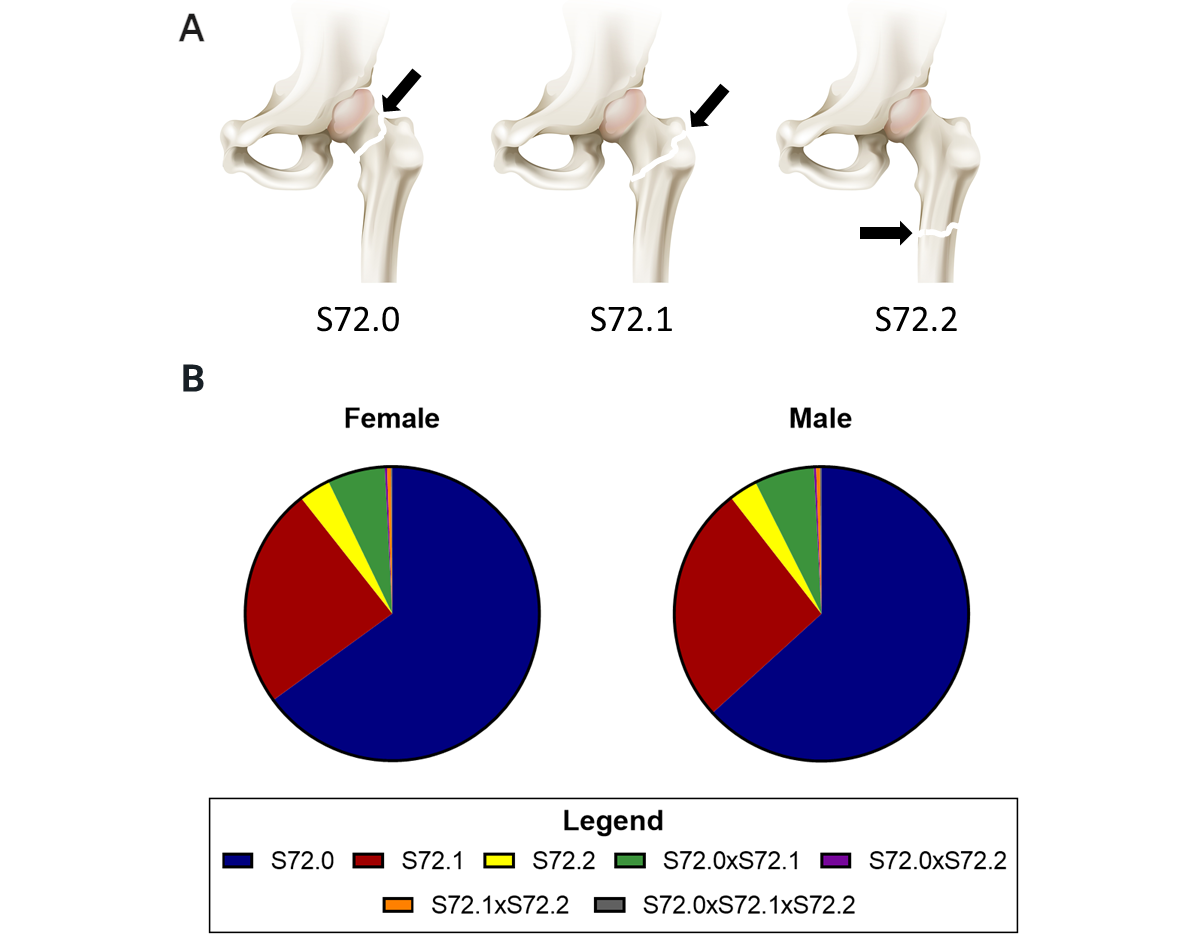
An anatomical depiction of the hip fracture codes and the percentage of hip fractures in patients 60 to 89 years of age with only 1 hip fracture event categorized by single, and combinations of, ICD-10 codes. Each hip fracture type is visually represented (A). As evident in the pie charts (B), the majority of patients with a single event of hip fracture broke the head or neck of the femur (S72.0). ICD-10: *International Classification of Diseases, Tenth Revision.*.

A total of 1,100,871 patients (758,995 [68.94%] female, 341,876 [31.06%] male) from 60 to 89 years of age with hip fracture codes of S72.0, S72.1, or S72.2 (Figure 1A), combinations thereof, or repeated fractures were retrieved from the past 10 years. Notably, narrowing the database query to only 1 fracture event of any fracture type or combination identified a substantially smaller pool of 408,922 patients (279,131 [68.26%] female, 129,791 [31.74%] male; Table 1) but reduced the confounding variables for the examination of individuals deceased within 1 year postfracture. Because simultaneous fracture combinations were allowed in the queries for 1 fracture event but repeated fracture events of the same type were not allowed, this suggests that more than 60% of elderly patients with hip fracture sustain multiple, temporally separated hip fractures of the same type or in the same region, ipsilaterally or contralaterally, or that the same fracture is repeatedly charted.

The racial and ethnic profiles of the elderly individuals in the TriNetX database’s Diamond Network that experienced a single hip fracture event were largely unreported, perhaps because the network is derived, at least in part, from insurance claims. However, patient mapping by zip code suggests that these patients were widely distributed throughout the United States (Figure S3 of Multimedia Appendix 3). The majority of these patients suffered a fracture of the femur head and neck. The second- and third-most common hip fracture types were pertrochanteric femur fracture and subtrochanteric femur fracture, respectively (Figure 1B). Importantly, less than 8% of elderly patients presented with multiple simultaneous fractures during a hip fracture injury.

**Table 1:** Incidence and relative frequency of various fractures in the TriNetX Diamond Network separated by fracture code/code combinations in cohorts split by sex and age. Incidence entries include individuals deceased the same day as the fracture. Data on separated ICD-10^a^ codes can be found in Table S2 of Multimedia Appendix 1; codes were aggregated, as described in the header for the table below, and individuals were only allowed 1 event with the ICD-10 code or pooled codes specified in each column from 60 to 89 years of age (instead of summing the codes of Table S2 of Multimedia Appendix 1 in order to avoid counting the same patient more than once). The relative frequency of each fracture/fracture grouping within a sex and age cohort is shown in parentheses and is specific to the fracture groupings studied herein, and there is a small possibility that patients were counted multiple times if they suffered fractures across the specified types/combinations.

Fracture site	Female			Male		
	60-69 years	70-79 years	80-89 years	60-69 years	70-79 years	80-89 years
Total counts of fracture events of the types/combinations queried, N	569,140	523,840	460,720	279,924	224,722	156,491
Hip (S72.0-2), n (%)	54,801 (9.6)	93,041 (17.8)	131,289 (28.5)	34,237 (12.2)	45,731 (20.4)	49,823 (31.8)
Humerus (S42.2), n (%)	74,332 (13.1)	72,525 (13.8)	54,692 (11.9)	30,484 (10.9)	23,277 (10.4)	14,242 (9.1)
Regions near the hip (S32.0-6), n (%)	100,347 (17.6)	131,154 (25.0)	149,943 (32.5)	66,584 (23.8)	65,507 (29.2)	52,597 (33.6)
Nonhip regions of the femur (S72.3-4), n (%)	19,796 (3.5)	23,556 (4.5)	24,608 (5.3)	11,186 (4.0)	9303 (4.1)	6928 (4.4)
Knee and lower leg (S82.0-4), n (%)	99,551 (17.5)	71,298 (13.6)	39,601 (8.6)	50,272 (18.0)	30,695 (13.7)	13,427 (8.6)
Talus, malleoli, and foot (S82.5-6, S92.1, S92.3-5), n (%)	220,313 (38.7)	132,266 (25.2)	60,587 (13.2)	87,161 (31.1)	50,209 (22.3)	19,474 (12.4)

^a^ICD-10: *International Classification of Diseases, Tenth Revision.*

The incidence and relative frequency of each skeletal region suffering a 1-time fracture changed as individuals aged (Table 1). For individuals 60-69 years of age, the most commonly affected skeletal region with a single fracture event (of those studied) was the talus, malleoli, and foot. In contrast, the hip and regions near the hip were the most commonly affected areas with a single fracture event in 80-89-year-olds. Importantly, the likelihood that a fracture event would occur at the hip increased nearly 3-fold in 80-89-year-olds compared to 60-69-year-olds, while the relative frequency of fracture types distal to the hip either decreased or remained largely unchanged during aging. The incidence of 1-time fractures of the portion of the femur distal to the lesser trochanter (nonhip regions of the femur) remained considerably lower than other fracture groupings regardless of age.

The largely increased incidence of hip fractures during aging likely reflects age-dependent changes, such as those of lifestyle, bone quality (eg, cortical porosity) [28], bone mineral density [29,30], or a combination of these factors. Table S2 of Multimedia Appendix 1 further segments each of the fracture groupings into unique ICD-10 codes; it includes more patients than Table 1 since individuals in a cohort with a specific ICD-10 code were allowed to have other fractures that were previously blocked via grouping. Consequently, Table 1 is more likely to eliminate confounding factors associated with fracture risk (eg, increased risk of fractures from osteoporosis [31]), while Table S2 of Multimedia Appendix 1 has greater statistical power.

Although the relative frequency of the various fracture groupings typically varied with increasing age (Table 1), the 1-year postfracture mortality rates across all fracture groupings increased with age and were consistently greater in males than in females (Table 2). The latter observation held for every ICD-10 code when analyzed individually (as demonstrated in Table S3 in Multimedia Appendix 1) and reflects the underlying greater general mortality rate associated with older age and being male [32,33]. Across all cohorts, patients with hip fracture consistently showed greater 1-year mortality rates compared to all other fractures, regardless of whether the comparison was to other fracture groupings (Table 2), to unique ICD-10 codes (as outlined in Table S3 of Multimedia Appendix 1), or to propensity-score-matched patients of fracture codes and groupings, as described more later (Tables 3 and 4, as well as Table S4 of Multimedia Appendix 1).

**Table 2:** One-year postfracture mortality rates separated by sex and age. ICD-10^a^ codes were aggregated, and patients were only allowed 1 event with the ICD-10 code or pooled codes specified in each column from 60 to 89 years of age; same-day deaths were not included. One-year postfracture mortality rates for individual ICD-10 codes are summarized in Table S3 of Multimedia Appendix 1.

Fracture site	Female				Male		
	60-69 years	70-79 years	80-89 years	60-69 years		70-79 years	80-89 years
Hip (S72.0-2), n/N (%)	3573/54,775 (6.5)	8751/92,946 (9.4)	17,165/131,031 (13.1)	2859/34,213 (8.4)		6227/45,650 (13.6)	9743/49,673 (19.6)
Humerus (S42.2), n/N (%)	2089/74,308 (2.8)	3528/72,495 (4.9)	4908/54,640 (9.0)	1388/30,469 (4.6)		1910/23,259 (8.2)	2042/14,212 (14.4)
Regions near the hip (S32.0-6), n/N (%)	4664/100,310 (4.6)	9511/131,077 (7.3)	15,154/149,797 (10.1)	3961/66,555 (6.0)		6905/65,437 (10.6)	8385/52,495 (16.0)
Nonhip regions of the femur (S72.3-4), n/N (%)	985/19,785 (5.0)	1844/23,539 (7.8)	3146/24,562 (12.8)	653/11,183 (5.8)		921/9294 (9.9)	1164/6,909 (16.8)
Knee and lower leg (S82.0-4), n/N (%)	1907/99,535 (1.9)	2671/71,274 (3.7)	3216/39,571 (8.1)	1393/50,262 (2.8)		1528/30,680 (5.0)	1337/13,407 (10.0)
Talus, malleoli, and foot (S82.5-6, S92.1, S92.3-5), n/N (%)	2661/220,300 (1.2)	3307/132,242 (2.5)	3693/60,562 (6.1)	1839/87,149 (2.1)		2064/50,196 (4.1)	1692/19,464 (8.7)

^a^ICD-10: *International Classification of Diseases, Tenth Revision*.

Importantly, for each cohort, 1-year mortality rates following fractures of the talus, malleoli, and foot occurring in a single event (Table 2) closely aligned with recent reports of mortality rates in the general US population [32,33], suggesting that these fractures do not cause excess mortality in elderly patients. Consequently, we used these fractures as a baseline to estimate the general 1-year mortality risk, against which we assessed excess 1-year mortality risks resulting from the various fracture groupings. Overall, 1-year mortality rates following hip fracture ranged from 6.5% for 60-69-year-old females to 19.6% for 80-89-year-old males (Table 2). Relative to the estimated mortality rate in the general population, hip fractures increased 1-year mortality rates by 5%-7% for female patients and 6%-11% for male patients (Table 2), with the largest absolute increases in mortality rates paralleling increases in age. However, the largest relative change in 1-year mortality rates occurred among 60-69-year-old patients, with females and males at 5.4-fold and 4-fold greater mortality risk, respectively, following hip fracture compared to that estimated for the general population.

**Figure 2:**
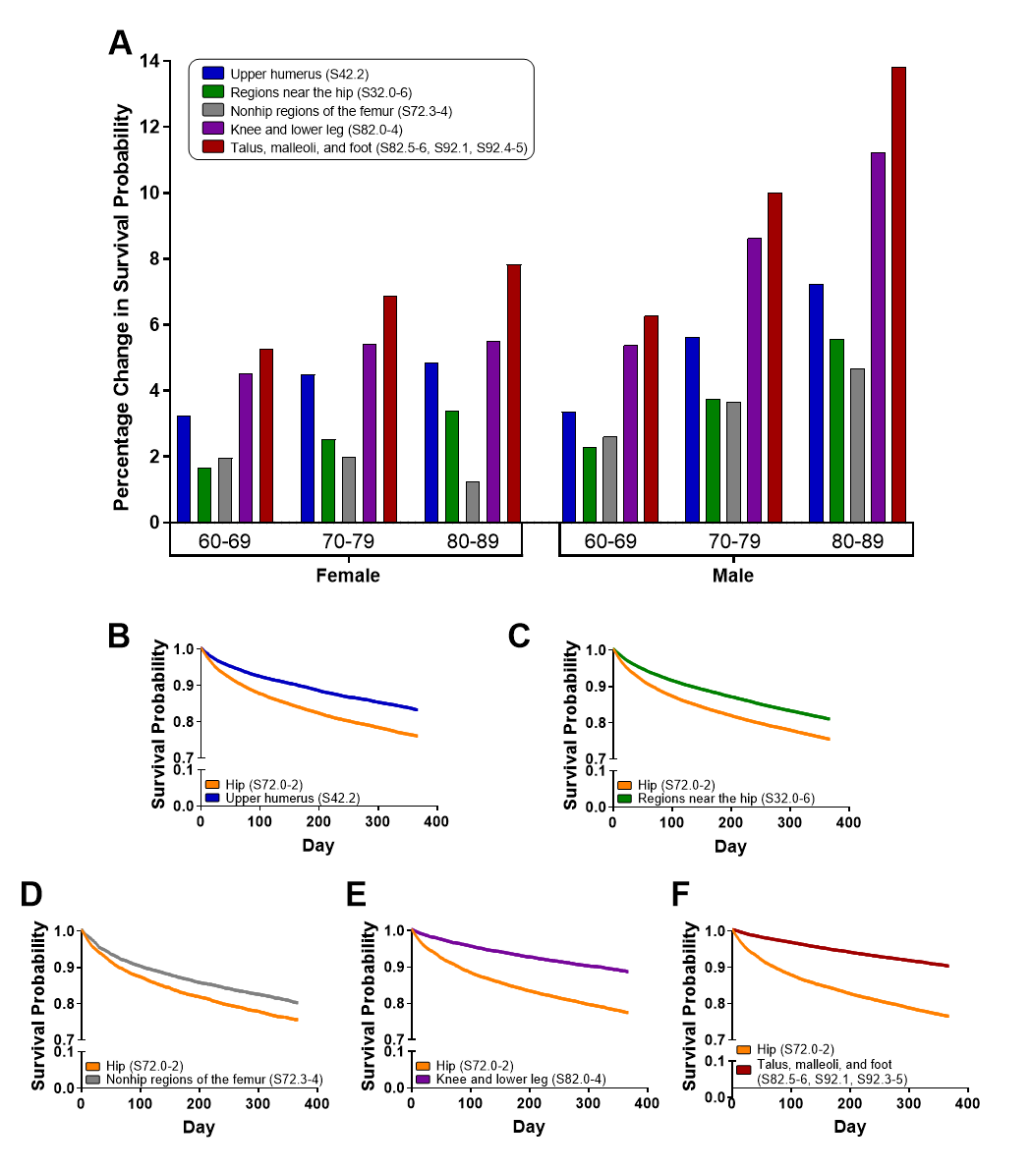
The changes to survival probability 1 year after several fracture types relative to propensity-score-matched hip fracture patients. Men from 80 to 89 years of age showed the largest absolute percentage change in survival rates across all fracture types relative to their propensity-score-matched patient cohorts with hip fracture. KM survival curves for men from 80 to 89 years of age with fractures of the hip compared to fractures of the upper humerus (B), regions near the hip (C), nonhip regions of the femur (D), knee and lower leg (E), and talus, malleoli, and foot (F). Hip fracture survival probability curves were not identical in each figure since each independent propensity score matching analysis for each comparison selected different groups of hip fracture patients. KM: Kaplan-Meier.

Rigorous KM and HR analyses of propensity-score-matched cohorts 1 year postfracture strongly supported the general population results (Tables 3 and 4, as well as Table S4 of Multimedia Appendix 1). Patients with hip fracture were propensity-score-matched to corresponding cohorts of patients with fractures of the upper humerus; regions near the hip; nonhip regions of the femur; knee and lower leg; or talus, malleoli, and foot. KM log-rank tests showed that hip fracture significantly decreased the 1-year postfracture survival probability relative to all other fracture groupings in each of the age and sex cohorts (Table 3). The percentage change in survival probability 1 year postfracture for each fracture grouping relative to the hip fracture grouping is shown in Figure 2A. The cohort with the largest absolute changes in 1-year survival rates across all fracture types was males 80-89 years of age (Figure 2B-F). Propensity-score-matched comparisons of the hip fracture group to each of the individual ICD-10 codes resulted in significantly lower survival probabilities for hip fracture relative to each other fracture type, with 3 exceptions: results were not significantly different for 60-69-year-old males with ischium or femur shaft fractures and for 80-89-year-old females with fractures of the lower end of the femur (Table S4 of Multimedia Appendix 1).

HRs calculated for hip fracture relative to all other fracture groupings exceeded 1 in all instances (Table 4). Importantly, the HRs for hip fractures relative to fractures of the talus, malleoli, and foot exceeded 3 in the 60-69- and 70-79-year-old female and male cohorts. Similarly, HRs established for hip fracture relative to each ICD-10 code exceeded 1 in all instances and exceeded 4.8 when hip fracture was compared to fracture(s) of the greater or lesser toe in 60-69-year-old females (Table S4 of Multimedia Appendix 1).

**Table 3:** Results from the KM^a^ analysis performed on data from 1 day to 1 year following fracture for the propensity-score-matched sex and age cohorts of patients with a hip fracture (individually or in combination: S72.0, S72.1, and S72.2) relative to the other fractures/fracture combinations specified within the table. Individuals deceased the same day as the fracture were not included in the patient cohorts. Only 1 hip fracture or other respective fracture-type event specified for each row was allowed from 60 to 89 years of age. Analyses performed on separated ICD-10^b^ codes can be found in Table S4 of Multimedia Appendix 1.

Age group (years)			Hip fracture cohort statistics				Other fracture cohort statistics					Log-rank test		
			Patients with outcome/patients in cohort, n/N (%)		Probability of 1-year survival (determined by KM curve), %		Patients with outcome/patients in cohort, n/N (%)		Probability of 1-year survival (determined by KM curve), %		χ^2^ (*df*)			*P* value^c^
**Upper humerus (S42.2)**														
	60-69, female	3234/50,849 (6.4)		92.9		1760/51,154 (3.4)		96.2		481.0 (1)			*<.001*	
	70-79, female	5773/63,408 (9.1)		89.8		3283/64,022 (5.1)		94.3		81.6 (1)			*<.001*	
	80-89, female	7027/52,099 (13.5)		84.7		4956/52,986 (9.4)		89.5		523.0 (1)			*<.001*	
	60-69, male	1986/24,947 (8.0)		91.1		1253/25,135 (5.0)		94.5		192.8 (1)			*<.001*	
	70-79, male	2945/21,583 (13.6)		84.7		1912/21,906 (8.7)		90.4		300.5 (1)			*<.001*	
	80-89, male	2809/13,227 (21.2)		75.7		2065/13,585 (15.2)		82.9		204.1 (1)			*<.001*	
**Regions near the hip (S32.0-6)**														
	60-69, female	3553/53,217 (6.7)		92.6		2793/53,489 (5.2)		94.2		111.9 (1)			*<.001*	
	70-79, female	8283/84,349 (9.8)		89.0		6514/85,097 (7.7)		91.5		293.5 (1)			*<.001*	
	80-89, female	14,975/107,520 (13.9)		84.1		12,176/109,465 (11.1)		87.5		511.1 (1)			*<.001*	
	60-69, male	2834/32,955 (8.6)		90.2		2205/33,177 (6.6)		92.5		100.0 (1)			*<.001*	
	70-79, male	5877/40,940 (14.4)		83.7		4653/41,515 (11.2)		87.4		226.7 (1)			*<.001*	
	80-89, male	8381/38,966 (21.5)		75.1		6829/40,065 (17.0)		80.7		358.6 (1)			*<.001*	
**Nonhip regions of the femur (S72.3-4)**														
	60-69, female	1326/19,457 (6.8)		92.5		990/19,485 (5.1)		94.4		56.5 (1)			*<.001*	
	70-79, female	2239/22,914 (9.8)		89.1		1860/22,973 (8.1)		91.1		46.6 (1)			*<.001*	
	80-89, female	3367/23,357 (14.4)		83.6		3171/23,481 (13.5)		84.9		12.0 (1)			*<.001*	
	60-69, male	890/10,866 (8.2)		90.7		656/10,924 (6.0)		93.3		43.1 (1)			*<.001*	
	70-79, male	1196/8879 (13.5)		84.9		927/8991 (10.3)		88.5		50.0 (1)			*<.001*	
	80-89, male	1397/6445 (21.7)		75.2		1170/6533 (17.9)		79.9		39.5 (1)			*<.001*	
**Knee and lower leg (S82.0-4)**														
	60-69, female	3423/52,623 (6.5)		92.8		1311/53,038 (2.5)		97.3		1051.7 (1)			*<.001*	
	70-79, female	5528/62,697 (8.8)		90.1		2570/63,403 (4.1)		95.5		1292.2 (1)			*<.001*	
	80-89, female	4973/37,797 (13.2)		85.1		3249/38,529 (8.4)		90.6		518.2 (1)			*<.001*	
	60-69, male	2517/31,105 (8.1)		90.8		1071/31,392 (3.4)		96.2		675.8 (1)			*<.001*	
	70-79, male	3475/26,988 (12.9)		85.4		1479/27,542 (5.4)		94.0		1032.7 (1)			*<.001*	
	80-89, male	2489/12,472 (19.9)		77.1		1357/12,934 (10.5)		88.3		538.7 (1)			*<.001*	
**Talus, malleoli, and foot (S82.5-6, S92.1, S92.3-5)**														
	60-69, female	3592/53,725 (6.7)		92.5		1082/54,228 (2.0)		97.8		1505.5 (1)			*<.001*	
	70-79, female	7258/78,836 (9.2)		89.7		2517/80,084 (3.1)		96.5		2741.9 (1)			*<.001*	
	80-89, female	7390/56,166 (13.2)		85.2		3669/57,850 (6.3)		93.0		1754.0 (1)			*<.001*	
	60-69, male	2754/32,621 (8.4)		90.4		988/33,094 (3.0)		96.7		976.0 (1)			*<.001*	
	70-79, male	4606/34,302 (13.4)		84.8		1655/35,247 (4.7)		94.8		1802.9 (1)			*<.001*	
	80-89, male	3687/17,652 (20.9)		76.1		1687/18,540 (9.1)		90.0		1201.9 (1)			*<.001*	

^a^KM: Kaplan-Meier.

^b^ICD-10: *International Classification of Diseases, Tenth Revision.*

^c^Statistically significant (*P*<.05) results are italicized.

**Table 4:** Results from the HR^a^ analysis performed on data from 1 day to 1 year following fracture for the propensity-score-matched sex and age cohorts of patients with a hip fracture (individually or in combination: S72.0, S72.1, and S72.2) relative to the other fractures/fracture combinations specified within the table. Individuals deceased the same day as the fracture were not included in the patient cohorts. Only 1 hip fracture or other respective fracture-type event specified for each row was allowed from 60 to 89 years of age. Analyses performed on separated ICD-10^b^ codes can be found in Table S4 of Multimedia Appendix 1.

Age group (years)		HR (95% CI)		χ^2^ (*df*)		*P* value^c^
**Upper humerus (S42.2)**						
	60-69, female	1.89 (0.167-21.488)	23.7 (1)		*<.001*	
	70-79, female	1.85 (0.172-19.934)	24.4 (1)		*<.001*	
	80-89, female	1.52 (0.16-14.522)	51.6 (1)		*<.001*	
	60-69, male	1.64 (0.145-18.584)	0.9 (1)		.36	
	70-79, male	1.66 (0.164-16.752)	18.5 (1)		*<.001*	
	80-89, male	1.51 (0.181-12.564)	14.7 (1)		*<.001*	
**Regions near the hip (S32.0-6)**						
	60-69, female	1.31 (0.12-14.22)	2.4 (1)		.12	
	70-79, female	1.33 (0.13-13.493)	29.7 (1)		*<.001*	
	80-89, female	1.32 (0.144-12.017)	124.1 (1)		*<.001*	
	60-69, male	1.33 (0.129-13.651)	0.8 (1)		.36	
	70-79, male	1.34 (0.146-12.334)	22.7 (1)		*<.001*	
	80-89, male	1.36 (0.176-10.513)	57.3 (1)		*<.001*	
**Nonhip regions of the femur (S72.3-4)**						
	60-69, female	1.37 (0.127-14.732)	2.3 (1)		.13	
	70-79, female	1.24 (0.123-12.466)	1.5 (1)		.22	
	80-89, female	1.09 (0.123-9.64)	0.04 (1)		.85	
	60-69, male	1.40 (0.134-14.564)	2.0 (1)		.16	
	70-79, male	1.36 (0.146-12.682)	6.8 (1)		*<.01*	
	80-89, male	1.28 (0.163-10.105)	3.9 (1)		*.049*	
**Knee and lower leg (S82.0-4)**						
	60-69, female	2.75 (0.243-31.034)	37.1 (1)		*<.001*	
	70-79, female	2.30 (0.214-24.769)	56.8 (1)		*<.001*	
	80-89, female	1.66 (0.172-16.08)	47.7 (1)		*<.001*	
	60-69, male	2.50 (0.23-27.213)	12.4 (1)		*<.001*	
	70-79, male	2.61 (0.261-26.143)	41.7 (1)		*<.001*	
	80-89, male	2.15 (0.247-18.681)	35.9 (1)		*<.001*	
**Talus, malleoli, and foot (S82.5-6, S92.1, S92.3-5)**						
	60-69, female	3.53 (0.311-39.961)	51.4 (1)		*<.001*	
	70-79, female	3.15 (0.291-34.06)	116.8 (1)		*<.001*	
	80-89, female	2.28 (0.23-22.52)	210.4 (1)		*<.001*	
	60-69, male	3.01 (0.279-32.493)	18.7 (1)		*<.001*	
	70-79, male	3.17 (0.315-31.767)	126.0 (1)		*<.001*	
	80-89, male	2.66 (0.3-23.659)	94.1 (1)		*<.001*	

^a^HR: hazard ratio.

^b^ICD-10: *International Classification of Diseases, Tenth Revision*.

^c^Statistically significant (*P*<.05) results are italicized.

### Dementia and Type 2 Diabetes Comorbidities

The codes used to identify elderly patients in the TriNetX database diagnosed with either dementia, T2D, or both comorbidities identified approximately 1.6 million, 16.8 million, or 0.66 million individuals, respectively (Figure S2 of Multimedia Appendix 3). Subsequently, we explored general incidence of the fracture groupings in combination with the comorbidities in our age range of interest (Table 5) and the relative frequency of the various fracture groupings in patients with dementia (Figure S3A of Multimedia Appendix 3) or T2D (Figure S3B of Multimedia Appendix 3) across the sex and age cohorts of interest. A dementia diagnosis clearly shifted the fracture type away from talus, malleoli, and foot fracture toward hip fracture in all cohorts and toward fracture of regions near the hip in some cohorts (Figure S3A of Multimedia Appendix 3). In contrast, a T2D diagnosis had a much smaller impact, shifting the fracture type slightly away from hip fracture toward talus, malleoli, and foot fracture in some cohorts, although these effects were minimal (Figure S3B of Multimedia Appendix 3). Similar to the general population cohorts, patients with hip fracture and dementia displayed greater mortality rates compared to other fracture groupings (Table 6) and all other ICD-10 codes studied (Table S5 of Multimedia Appendix 1).

**Table 5:** General incidence and relative frequency of various fracture types/combinations with dementia/T2D^a^ in patients 60-89 years of age. Dementia diagnosis was allowed from any time before fracture to 1 year postfracture and was specified as any of the following ICD-10^b^ codes or a combination thereof: vascular dementia, F01; dementia due to Alzheimer disease, G30; or dementia with Lewy bodies, G31.83. T2D was required to be recorded within 6 months prior to fracture to 1 year postfracture and was specified with ICD-10 code E11. ICD-10 codes were aggregated as described in the header for this table, and patients were only allowed 1 event with the code or pooled codes specified in each column from 60 to 89 years of age. Incidence entries include individuals deceased the same day as the fracture. The relative frequency of each fracture/fracture grouping is shown in parentheses and is specific to the fracture groupings studied herein and, as with Table 1, there is a small possibility that patients were counted multiple times if they suffered fractures across the specified types/combinations.

Total counts of fracture events of the types/combinations queried in combination with dementia/T2D	Hip (S72.0-2), n (%)	Humerus (S42.2), n (%)	Regions near the hip (S32.0-6), n (%)	Nonhip regions of the femur (S72.3-4), n (%)	Knee and lower leg (S82.0-4), n (%)	Talus, malleoli, and foot (S82.5-6, S92.1, S92.3-5), n (%)
With dementia (N=112,113)	37,084 (33.1)	12,625 (11.3)	34,211 (30.5)	6425 (5.7)	9070 (8.1)	12,698 (11.3)
With T2D (N=594,711)	100,804 (17.0)	72,748 (12.2)	146,301 (24.6)	28,353 (4.8)	91,376 (15.4)	155,129 (26.1)

^a^T2D: type 2 diabetes.

^b^ICD-10: *International Classification of Diseases, Tenth Revision*.

**Table 6:** One-year postfracture mortality rates in patients with dementia (specifically, any of the following ICD-10^a^ codes or a combination thereof: vascular dementia, F01; dementia due to Alzheimer disease, G30; or dementia with Lewy bodies, G31.83) or T2D^b^ (E11). A dementia diagnosis was allowed from any time before fracture to 1 year postfracture, while a T2D diagnosis was required to be recorded within 6 months prior to fracture to 1 year postfracture. Data on separated ICD-10 codes can be found in Table S5 of Multimedia Appendix 1; ICD-10 codes were aggregated as described in the header for this table, and patients were only allowed 1 event with the ICD-10 code or pooled codes specified in each column from 60 to 89 years of age. Same-day deaths were not included for this analysis.

Age group (years)		Hip (S72.0-2), n/N (%)	Humerus (S42.2), n/N (%)	Regions near the hip (S32.0-6), n/N (%)	Nonhip regions of the femur (S72.3-4), n/N (%)	Knee and lower leg (S82.0-4), n/N (%)	Talus, malleoli, and foot (S82.5-6, S92.1, S92.3-5), n/N (%)
**With dementia**							
	60-69, female	180/1117 (16.1)	69/741 (9.3)	177/1391 (12.7)	42/307 (13.7)	76/804 (9.5)	85/1236 (6.9)
	70-79, female	1213/6512 (18.6)	390/3009 (13.0)	945/6590 (14.3)	210/1270 (16.5)	240/2237 (10.7)	298/3280 (9.1)
	80-89, female	3955/18,452 (21.4)	983/6031 (16.3)	2787/16,301 (17.1)	668/3383 (19.7)	591/3741 (15.8)	701/4877 (14.4)
	60-69, male	140/854 (16.4)	47/385 (12.2)	119/922 (12.9)	23/151 (15.2)	46/401 (11.5)	45/598 (7.5)
	70-79, male	813/3388 (24.0)	206/1015 (20.3)	673/3349 (20.1)	87/463 (18.8)	124/863 (14.4)	153/1264 (12.1)
	80-89, male	1916/6647 (28.8)	337/1429 (23.6)	1355/5608 (24.2)	206/838 (24.6)	194/1016 (19.1)	286/1431 (20.0)
**With T2D**							
	60-69, female	1217/13,972 (8.7)	840/18,279 (4.6)	1640/24,610 (6.7)	460/5881 (7.8)	856/23,717 (3.6)	1246/49,752 (2.5)
	70-79, female	2728/23,917 (11.4)	1337/20,425 (6.5)	3234/34,770 (9.3)	756/7425 (10.2)	1751/25,936 (6.8)	1594/36,892 (4.3)
	80-89, female	3695/26,451 (14.0)	1295/12,935 (10.0)	3786/31,507 (12.0)	882/6351 (13.9)	10,83/10,225 (10.6)	1315/16,585 (7.9)
	60-69, male	1033/9552 (10.8)	604/8635 (7.0)	1400/18,454 (7.6)	255/3213 (7.9)	608/13,575 (4.5)	941/26,241 (3.6)
	70-79, male	2146/14,099 (15.2)	868/8087 (10.7)	2698/21,630 (12.5)	398/3264 (12.2)	1039/13,445 (7.7)	1116/18,562 (6.0)
	80-89, male	2570/12,659 (20.3)	668/4323 (15.5)	2618/15,195 (17.2)	385/2186 (17.6)	552/4428 (12.5)	745/7055 (10.6)

^a^ICD-10: *International Classification of Diseases, Tenth Revision*.

^b^T2D: type 2 diabetes.

For individuals with T2D and fracture, 1-year mortality results were similar to those of the general population. All cohorts with a T2D diagnosis exhibited a greater 1-year mortality rate after hip fracture relative to the other fracture groupings (Table 6) and other individual ICD-10 codes, with the exception of females with fracture of the lower end of the femur from 80 to 89 years of age (Table S5 of Multimedia Appendix 1).

**Figure 3:**
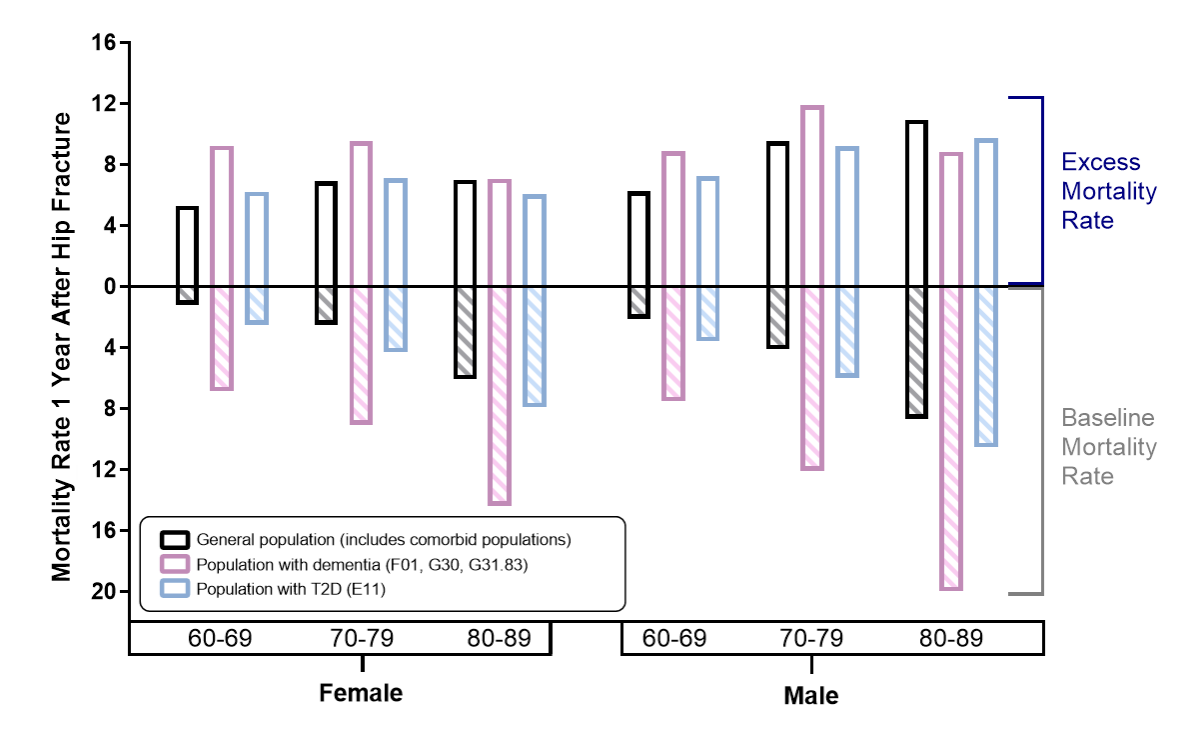
The effects of dementia and T2D in combination with hip fracture on mortality. To establish whether these comorbidities combined with hip fracture exacerbate the mortality rate beyond simply additive effects, the percentage of individuals with the comorbidity of interest deceased 1 year postfracture of the talus, malleoli, and foot was superimposed onto the 1-year mortality rate following hip fracture with the respective comorbidity (this was used as a representation of the baseline mortality rate; for the general population no comorbidities were specified, and it included individuals with the comorbidities of the other populations). Results showed that hip fracture was synergistic with dementia in younger cohorts but was either additive or less than additive for older cohorts with dementia and nearly all cohorts with T2D. T2D: type 2 diabetes.

Collectively, these data determined the extent that dementia or T2D as comorbidities exacerbate 1-year postfracture mortality rates. The comorbidities of hip fracture and dementia in the 60-69- and 70-79-year-old cohorts resulted in greater excess 1-year mortality rates compared to hip fracture in the general population (Figure 3). However, T2D combined with hip fracture did not show a similar exacerbation, and 1-year mortality rates for hip fracture with a T2D comorbidity were typically additive (equal to the combination of the baseline mortality rate and the mortality rate due to hip fracture) or less than additive. Instances of less-than-additive effects suggest the possibility of a mutual cause of mortality or that the care of the comorbidity may reduce the hip fracture–related mortality risk.

## Discussion

### Potential Interpretations and Implications

Hip fractures confer a uniquely increased mortality risk relative to all other studied fracture groupings. From the upper humerus data, we inferred that the increased mortality is not the result of the fracture occurring at an appendage-torso juncture, and the remainder of comparisons exhibited generally increasing differences in mortality rates as the fracture site became more distal to the hip. Because more peripheral fractures are expected to have less of an impact on mobility, perhaps requiring a boot or a scooter instead of a wheelchair, these data suggest that mobility may play a critical role in mortality risk.

Given that the differences in mortality rates remained when patients were propensity-score-matched across cohorts, this suggests that there is likely 1 or more underlying hip-specific mechanisms. The comorbidity of dementia that was identified herein as acting synergistically with hip fracture to exacerbate mortality rates could not have been the sole driving force behind these increased mortality rates, since it was not synergistic at older ages where the percentage change in survival probability between hip fracture and talus, malleoli, and foot fracture was the greatest. As mentioned previously, 1 factor that might influence these observed differences in 1-year postfracture mortality rates is mobility, as it is common to not regain prefracture mobility following hip fracture [34-37]. This greater mortality risk associated with impaired mobility may stem from changes to self-care factors, such as the ability to acquire help immediately following a fall, or changes to the blood flow dynamics, since circulatory system disease has been identified as a leading cause of death in patients after hip fracture [38]. Additionally, greater proportions of daily sitting time have been associated with increased all-cause mortality risk [39]. Consequently, restoring mobility should be a treatment priority to reduce the mortality risk associated with hip fracture and other mobility-impairing fractures. Additionally, the differences in mortality rate between hip fracture and other types of fracture might result from differences in tensile strength [40], bone density [41], or bone quality; for instance, the femoral head is less isotropic but has consistently greater trabecular bone volume than the humeral head [42]. Together, this strongly supports future investigations to identify unique behavioral and cellular mechanisms occurring following hip fracture.

Exploration of dementia as a comorbidity revealed that fracture combined with dementia substantially exacerbates the mortality rate in younger elderly cohorts. This suggests that the increased 1-year mortality rates observed in patients with dementia and fracture may arise from synergistic mechanisms, whether cellular or behavioral in nature. The apparent lack of excess mortality associated with T2D as a comorbidity to fracture aligns with studies that reported no differences in 1-year mortality rates in populations of individuals that included those with type 1 diabetes (T1D) and T2D [43,44].

This work extends previous studies that have attempted to distinguish comorbidity-related deaths from those brought about directly by hip fracture. Previous studies have attributed 17%-32% of all hip fracture–associated deaths directly to the fracture event after subtracting out the comorbidities [45]. In contrast, this work indicates that hip fracture increases the 1-year mortality risk in the general elderly population by approximately 2- to 5-fold. In elderly patients with dementia, hip fracture approximately doubles the 1-year mortality risk for all but the oldest cohorts of patients. Our study further established that this excess mortality is not a consequence of any fracture type but is instead directly related to fracture location, with fractures of the hip associated with the greatest increases in the 1-year mortality rate.

### Limitations

Despite the rigor of this work, several confounding variables remain. The TriNetX database, and analogous databases developed from EMRs, has difficulty accounting for patients that leave the health care system, as well as patients that are inaccurately diagnosed or whose diagnosis is later changed. Patients may move into a health care system with incomplete records or transfer between health care systems that both import data to TriNetX’s Diamond Network, the latter of which could lead to counting a patient more than once. Moreover, in TriNetX, propensity score matching can only be performed through the day of an event, because testing statistics on survival requires the event to be the fracture event, and we could not propensity-score-match based on how individuals were treated or diagnosed postfracture.

Although same-day deaths, which accounted for less than 2% of the deaths that occurred within 1 year of hip fracture, were removed, we did not explore whether the trauma event(s) inciting or associated with hip fracture resulted in more extensive damage to the surrounding area that might acutely increase the risk of mortality. Low preoperative hemoglobin concentration and excessive blood loss during surgery are both linked to increased mortality rates [46], and patients with hip fracture and delayed surgical intervention present with significant blood loss over the days following hospital admission but before surgery [47]. Finally, a statistical limitation was the lack of corrections for multiple comparisons in the propensity-score-matched results, which were left out because this was an exploratory analysis and should be followed up with a prospective observational trial.

### Conclusion

Hip fracture results in a greater 1-year mortality rate relative to the upper humerus and other fracture types/groupings of regions near and below the hip. This increased risk remains when cohorts are propensity-score-matched across a large number of characteristics, suggesting that this vulnerability is specific to this particular fracture type. Furthermore, the data herein established that dementia acts synergistically with hip fracture to exacerbate mortality rates in younger populations, but T2D does not appear to impact the mortality rate beyond an additive effect of the risks conferred by T2D and hip fracture independently. The data strongly suggest the necessity of future studies to explore unique elements of hip fracture events and therapeutic options targeting this fracture type specifically.

## Appendix

Multimedia Appendix 1 The Excel file containing Tables S1-S5 referenced in the manuscript.

Multimedia Appendix 2 Additional details on propensity score matching methods.

Multimedia Appendix 3 The PDF containing Figures S1-S3 referenced in the manuscript.

## Abbreviations

EMR: electronic medical record
HR: hazard ratio
ICD-10: *International Classification of Diseases, Tenth Revision*
KM: Kaplan-Meier
T1D: type 1 diabetes
T2D: type 2 diabetes
