# Supplementary material for: One-Year Postfracture Mortality Rate in Older Adults With Hip Fractures Relative to Other Lower Extremity Fractures: Retrospective Cohort Study
Source: JMIR Aging. 2022 Mar 16;5(1):e32683. doi: 10.2196/32683 (PMC8968577; doi:10.2196/32683)
Supplement: Multimedia Appendix 2 [file aging_v5i1e32683_app2.docx]

**Additional Details on Propensity Score Matching Methods**

Within TriNetX, propensity scores are matched by first computing a score for each characteristic category (covariate) for every patient within both cohorts and, when relevant, an outcome^1^. Covariates are binary, categorical, or continuous, and the outcome in this study was the term “deceased,” which was examined 1 day to 1 year following a fracture diagnosis. After establishing the outcome and a value for each covariate for each patient, the patients’ covariate values are used to create a matrix for each cohort (patients across the rows and covariates across the columns). Logistic regression is then performed on the pooled cohort matrices to assign the probability that a patient, based on their covariate values, belongs in the second cohort assigned in the analyses. This probability value is the propensity score, which ranges from 0 to 1. In the current study, the first cohort assigned was the hip fracture cohort. Hence, the second cohort was any other type of fracture or combination of fractures analyzed. Starting with a patient in the smaller cohort, the TriNetX algorithm identifies the most similar propensity score (within a range) in the larger cohort, and pairwise matches patients until each patient in the smaller cohort is matched. In the event that there are not appropriately similar propensity scores in both cohorts, then the patient is removed from the analysis.

1. In compare outcomes, how are patients matched when balancing cohorts? 2021. https://support.trinetx.com/hc/en-us/articles/360011978033-In-compare-outcomes-how-are-patients-matched-when-balancing-cohorts-
