# Supplementary material for: One-Year Postfracture Mortality Rate in Older Adults With Hip Fractures Relative to Other Lower Extremity Fractures: Retrospective Cohort Study
Source: JMIR Aging. 2022 Mar 16;5(1):e32683. doi: 10.2196/32683 (PMC8968577; doi:10.2196/32683)
Supplement: Multimedia Appendix 3 [file aging_v5i1e32683_app3.pdf]

A. Females

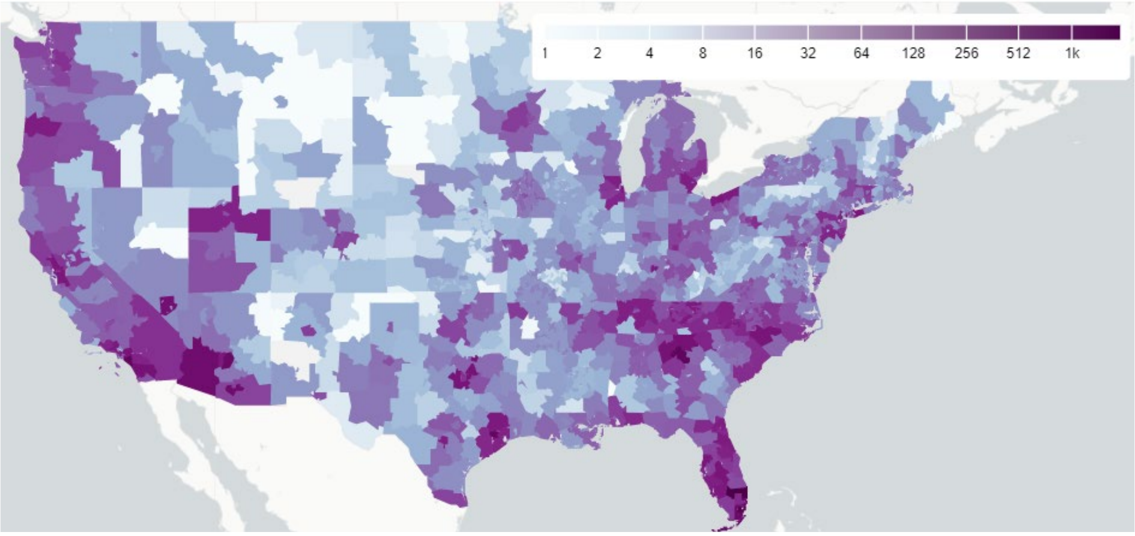

B. Males

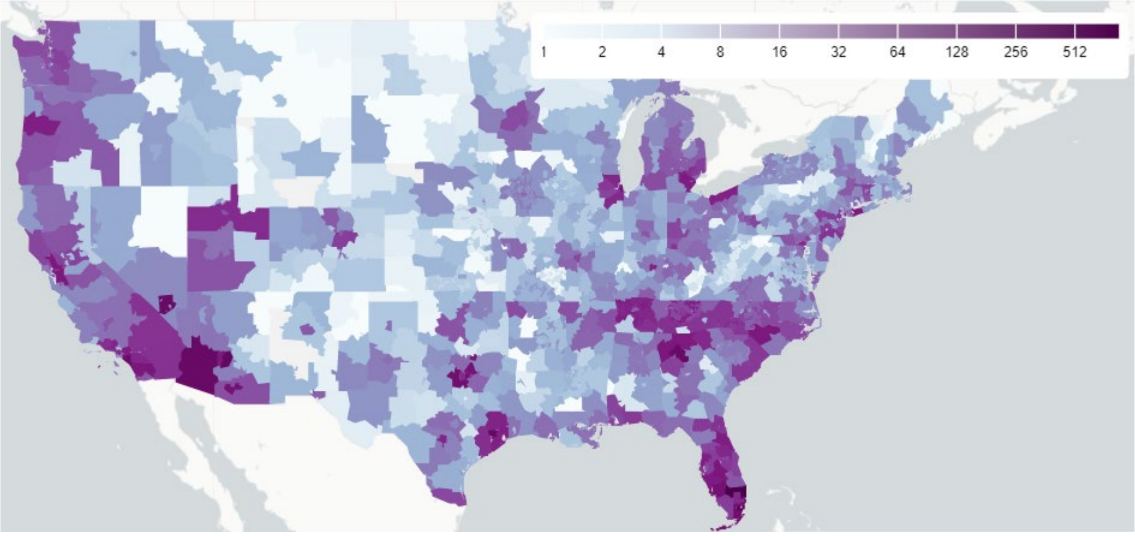

Figure S1. Patient density for those 60 to 89 year of age with 1 hip fracture event mapped by 3-digit zip code. Female (**A**) and male (**B**) patients in TriNetX's Diamond network. Maps were generated by TriNetX with assistance from Leaflet, OpenStreetMap contributors, CartoDB, and 2018 ZIP code data from Caliper/Maptitude. A total of 80,032 female patients were unable to be mapped while 35,992 male patients were unable to be mapped; patients in Alaska, Hawaii, and US territories are not pictured.

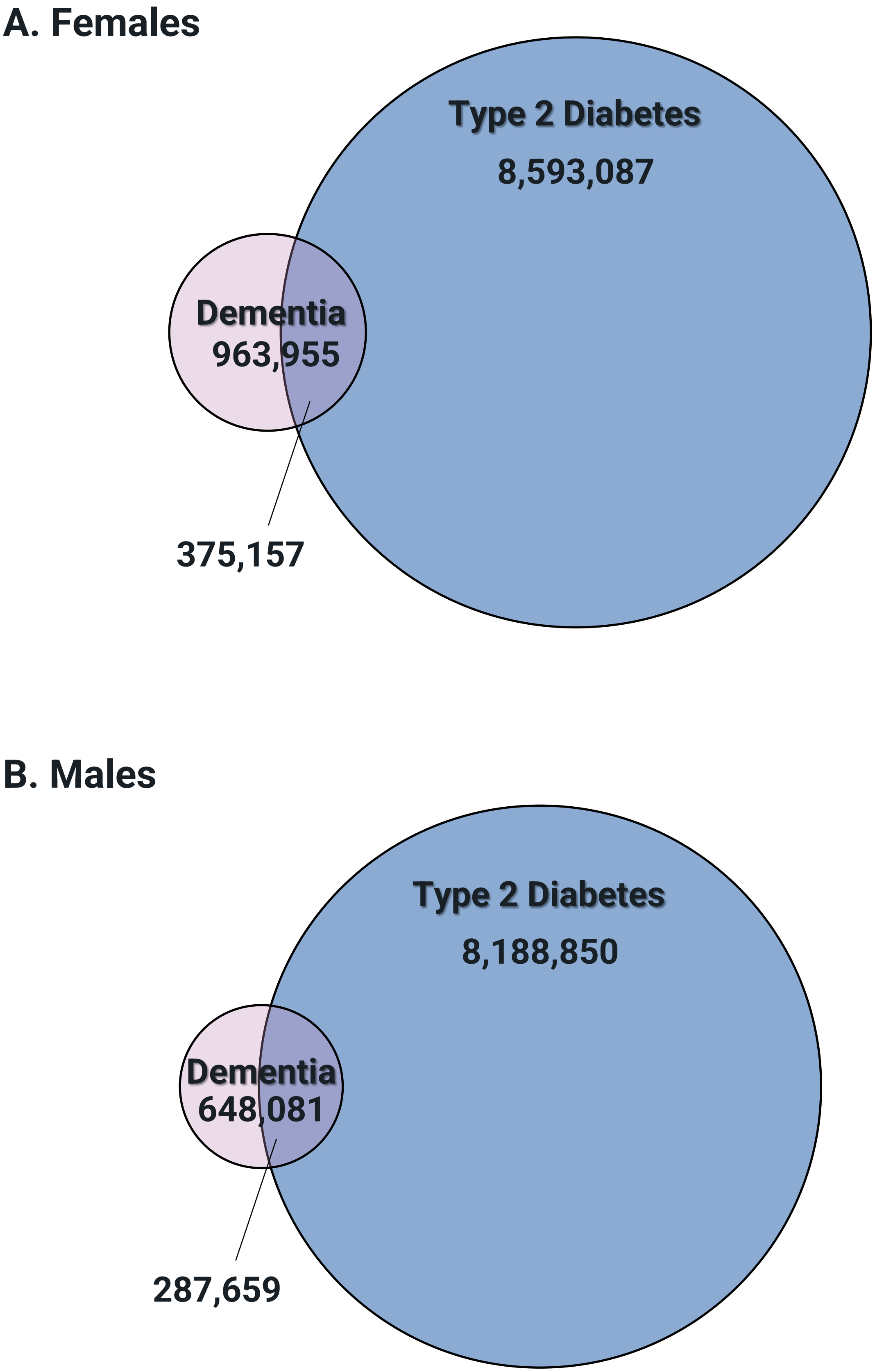

Figure S2. Populations affected by comorbidities of interest. Numbers were obtained from TriNetX's Diamond network for the female and male individuals diagnosed with dementia (F01, G30, G31.83), Type 2 Diabetes (E11), or both from 60 to 90 years of age, during the past decade (without time constraints for diagnoses). The Venn diagram was made using BioVenn[1].

1. Hulsen, T., de Vlieg, J., & Alkema, W. (2008). BioVenn - a web application for the comparison and visualization of biological lists using area-proportional Venn diagrams. BMC Genomics, 9, 488. https://doi.org/10.1186/1471-2164-9-488

A

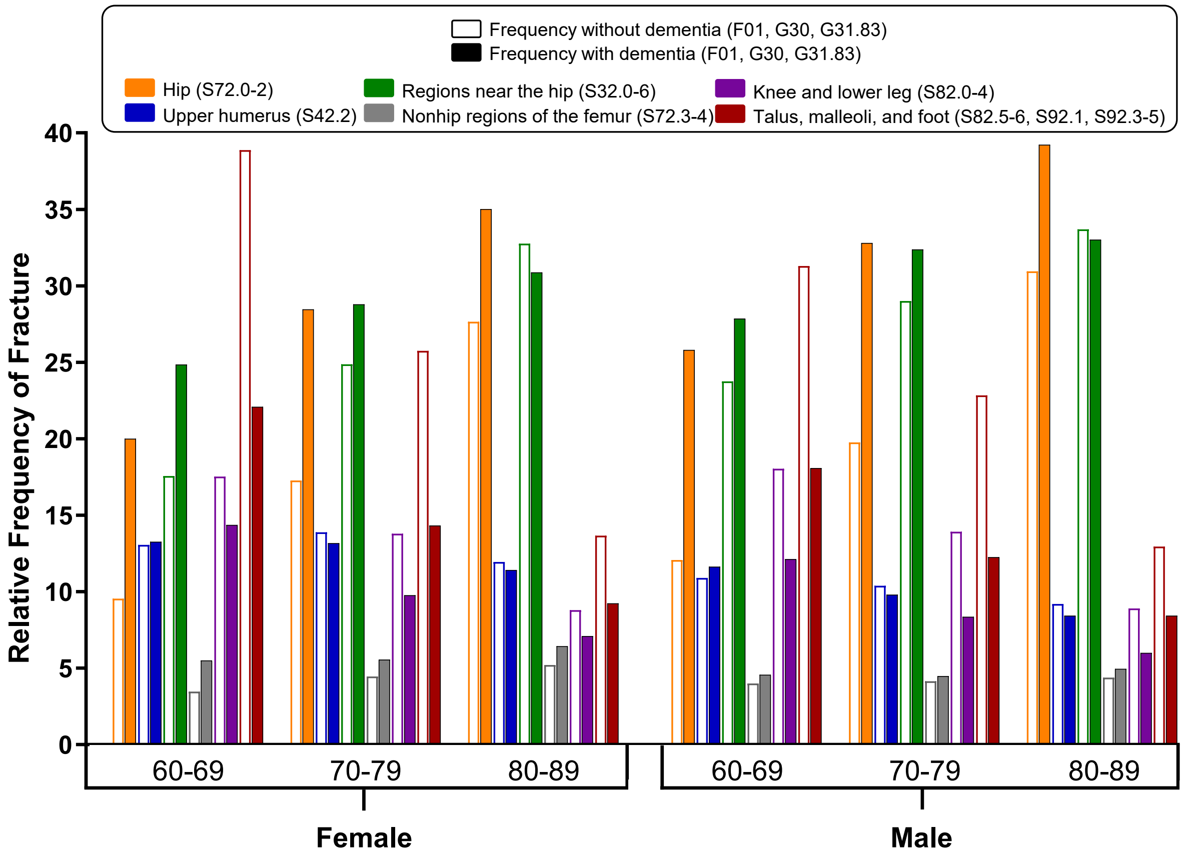

B

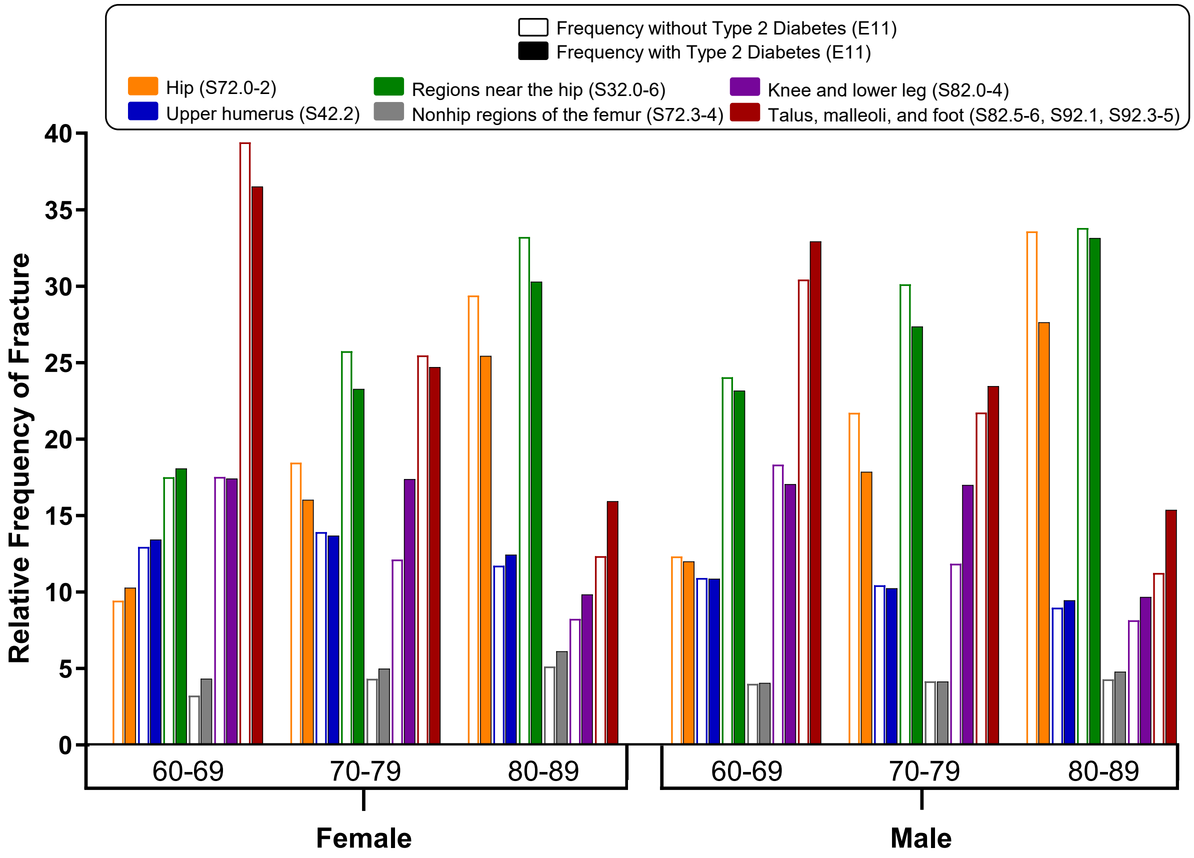

Figure S3. Relative frequency of fracture for patients with the comorbidities of interest. A diagnosis of dementia (diagnosis anytime before fracture through 1-year postfracture) was associated with a consistently increased relative frequency of hip fracture in all age/sex cohorts and a decreased relative frequency of talus, malleoli, and foot fractures (**A**). On the contrary, a diagnosis of Type 2 diabetes (diagnosis 6 months prior to fracture through 1-year postfracture) was not associated with dramatic changes to the relative frequency of hip fracture, or any other type of fracture (**B**).
